# The Subjective Well-being and Health-Related Quality of Life of Australian Adults with Increased Intestinal Permeability and Associations with Treatment Interventions

**DOI:** 10.1089/acm.2021.0202

**Published:** 2021-12-03

**Authors:** Bradley Leech, Erica McIntyre, Amie Steel, David Sibbritt

**Affiliations:** ^1^ Faculty of Health, Australian Research Centre in Complementary and Integrative Medicine, University of Technology Sydney, Ultimo, Australia.; ^2^ Institute for Sustainable Futures, University of Technology Sydney, Ultimo, Australia.

**Keywords:** intestinal permeability, intestinal barrier dysfunction, subjective wellbeing, health-related quality of life

## Abstract

***Objective:*** The integrity and function of the gastrointestinal system is important in disease prevention and management. This study aims to describe the management methods used by Australian adults with suspected increased intestinal permeability (IP) and the association with subjective wellbeing (SWB) and health-related quality of life (HRQoL).

***Design and Setting:*** Cross-sectional survey of Australian adults diagnosed with IP or have suspected (undiagnosed) IP.

***Outcome Measures:*** Questionnaire items investigating demographic characteristics, self-reported outcome of IP and treatment methods used to manage IP. Participants' HRQoL and SWB according to the 20-Item Short Form Health Survey (SF-20) and Personal Wellbeing Index-Adult (PWI-A) scale, respectively.

***Results:*** Participants (*n* = 589) frequently used dietary products (87.9%), dietary supplements (72.9%) and lifestyle therapies (54.6%) for managing IP. Participants had lower (i.e., worse) mean SWB scores for all domains compared to the Australian population (*p* < 0.001). The number of days IP reported to affect daily living was negatively correlated with SWB and HRQoL (*p* < 0.001). Participants that reported an improvement in their IP in the previous 12 months were more likely to be treated by a healthcare practitioner (OR = 2.04, *p* = 0.015), use dietary supplements (OR = 2.66, *p* = 0.003), participate in vigorous exercise (OR = 2.99, *p* < 0.001) and employ vagus nerve stimulation (OR = 3.10, *p* = 0.010). Conversely, they were less likely to consume gluten (OR = 0.35, *p* < 0.001) or use nonsteroidal anti-inflammatory drugs (OR = 0.35, *p* = 0.022). Self-reported improvement of IP (β = 10.70, *p* < 0.001) and use of dietary products (β = 12.12, *p* = 0.008) were predictors of a higher level of SWB.

***Conclusions:*** Altered IP may pose a greater health burden than previously thought, with poor SWB and HRQoL reported in Australian adults with self-reported IP. Our results highlight the potential clinical relevance and consequence of altered IP, providing the first indication of a possible relationship between altered IP and both SWB and HRQoL.

## Introduction

The health of the gastrointestinal system has become a target of interest for disease prevention.^1^ One specific gastrointestinal target area is the integrity of the intestinal barrier of the small intestine. During increased intestinal permeability (IP), the tight junction proteins between the cells of the small intestine disassemble in response to the protein zonulin.^2^ The single layer of epithelium cells in the small intestine contributes to the biochemical and physical barrier to the array of foreign pathogens, allergens, and other toxins.^3^ The prevalence of altered IP has been suggested to be 10%–87% in health conditions with a known association.^4^ During a loss of intestinal integrity, a cascade of reactions contributes to systemic symptoms and disease progression with the mitigation of zonulin that is suggested to inhibit or reduce disease onset.^5,6^ Although no defined symptoms of IP have been identified,^5,7^ a range of risk factors are known to be associated with altered IP.^5^ The clinical risk factors associated with IP provide a potential platform for treatment interventions and areas for further investigation.

The management of altered IP may involve the use or avoidance of dietary products (e.g., increasing dietary fiber, avoidance of gluten and alcohol), lifestyle therapies (e.g., stress management, vagus nerve stimulation), dietary supplements (e.g., vitamin A, probiotics, *Curcuma longa*, fish oil), and medication evaluation (e.g., avoidance of nonsteroidal anti-inflammatory drugs [NSAIDs] and antibiotics or the use of larazotide acetate).^6,8–10^ These methods are proposed to have multiple direct and indirect modulatory actions that regulate intestinal integrity.^8,9^ Many of the treatments used by practitioners for the management of IP have previously been shown to align with preclinical research.^9^ Although these treatment methods are frequently used in clinical practice, there still remains limited evidence for the effective management of altered IP. A broad health services research-based study may help identify the potential areas for further clinical trials.^11^

The clinical relevance and consequence of altered IP in clinical practice have recently been questioned,^12^ despite identified associations between IP and a wide range of health conditions.^4^ Questions regarding the clinical relevance and consequence of altered IP may stem from a low level of awareness and understanding regarding the potential effect of altered IP on individuals, especially their quality of life (QoL) and subjective well-being (SWB). QoL is an important contributor to overall disease burden alongside financial burden, mortality, and morbidity.^13,14^ Health-related quality of life (HRQoL) is a multidimensional concept that measures the impact of health status on QoL and includes mental, physical, emotional, and social functioning.^15^ In addition to HRQoL, a person's SWB—also referred to as life satisfaction—can be a determinant in quantifying the clinical relevance and consequence of ill health. The SWB is a multidimensional construct comprising cognitive and affective components that reflect an individual's appraisal of their satisfaction with their life.^16,17^ Understanding the SWB of individuals with particular health conditions may help identify populations with greater mortality risk^18^ and guide the development of targeted supportive interventions.

The impact of altered IP on individuals' HRQoL and SWB, and the treatments used in the management of IP, remains under-examined. As such, this study has two primary aims: to describe the SWB and HRQoL of Australian adults with suspected IP and to explore the treatment methods used by this population group.

## Materials and Methods

### Study design and setting

A cross-sectional study design using an online self-reported survey was deployed. Approval for the study was obtained from the Human Research Ethics Committees (HREC) of the University of Technology Sydney (ETH19-4012). The health-seeking behavior, views, and preferences of this study cohort have previously been published.^19^

### Participants and recruitment

Participants were recruited via social media platforms and a purpose-built webpage, with snowball sampling methods used. The authors shared the survey on their social media and known Facebook groups, such as Leaky Gut and Microbiome Support Group Australia. The survey was open for 2 months between September 2019 and November 2019. Eligibility questions asked participants whether they believe they have IP (self-diagnosed) or have been diagnosed with IP. To participate in the study, participants were also required to be 18 years of age or older, living in Australia, and have Internet access. The target population, although broad, represents an under-examined population group; as such, this study was designed to capture people with suspected IP or confirmed IP. As IP is suggested to be underdiagnosed, including participants who self-diagnose IP best reflects the target population and the patients who present to clinical practice for the treatment of IP.^7^ Survey responders with incomplete demographic characteristics, accounting for <5% of total data, were excluded from analysis.

### Survey and data collection

The online survey administered through the online platform *SurveyGizmo* utilized questionnaire items previously developed to investigate IP in Australia.^7,9^ The survey was pilot tested by four lay individuals to assess language clarity, with the required corrections made. The survey included four main domains: demographic characteristics, treatment methods for altered IP, SWB, and HRQoL.

### Demographic characteristics

The participants were asked about their gender, age, height, and weight from which body mass index (BMI) was calculated and categorized to underweight, healthy weight, overweight, and obese.^20^ The participants were further asked about their country of birth, the state or territory where they reside, and whether this was in an urban, rural, or remote location. The participant's income manageability was determined by how well they manage their household income, categorized as “difficult all the time,” “difficult some of the time,” “not too bad,” or “easy.”

### Self-reported outcome of increased IP

Two questions were asked to explore the potential severity of IP. First, participants were asked whether they believed their IP has become “better,” “worse,” or “no change” over the previous 12 months. Participants were then asked how many days a week does their IP affect their daily living with the option of 0–7 days.

### Treatment of increased IP

A selection of survey items involving dietary products, lifestyle therapies, dietary supplements, and medications that may either improve or exacerbate IP along with open-ended questions were used to document how frequently these methods were used. The frequency of use for dietary products, lifestyle therapies, dietary supplements, and medications were measured by using a six-point scale (“never,” “less than once a month,” “1–3 times a month,” “once a week,” “2–6 times a week,” “every day”). These treatment methods were further explored in relation to the person who prescribed the treatment, mainly the qualification of the practitioner or whether the treatment was self-prescribed.

### Subjective well-being

Participants' SWB was measured by using the Personal Well-being Index—Adult (PWI-A) scale—an instrument validated in Australian population samples.^21^ The PWI scale consisted of seven domains of satisfaction: *standard of living*, *personal health*, *achieving in life*, *personal relationships*, *personal safety*, *community-connectedness*, and *future security*.^21^ The PWI scoring system of each domain is reported on a 0–10 scale, with 0 representing no satisfaction at all and 10 being completely satisfied.

### Quality of life

The 20-Item Short Form Health Survey (SF-20) was used to measure participants' HRQoL.^22^ The SF-20 assesses six health domains: physical functioning (six questions), role functioning (two questions), social functioning (one question), mental health (five questions), current health perceptions (five questions), and bodily pain (one question).

### Statistical analyses

Data were exported to STATA^®^ 16 for statistical analyses. Variables were reported as means, standard deviations (SDs), 95% confidence intervals (CIs), or frequencies and percentages, where appropriate. Chi-square analysis was used to examine the association between two categorical variables, with Student's *t*-tests used for continuous variables across a binary variable. Analysis of variance was used to measure the difference between a continuous variable across a categorical variable. Spearman's correlation analysis was used to measure the correlation between the number of days that IP affects daily living, SWB, and HRQoL. Logistic and linear regression models were used when considering multiple factors. Variables associated with SWB, HRQoL, or the number of days that IP affects daily living—with a bivariate *p*-value <0.25^23^—were entered into the respective multivariate logistic or linear regression models, to adjust for potential confounders. A stepwise backward elimination process was then used to identify the most important independent predictors.

For analysis, participants' use of dietary products, lifestyle therapies, dietary supplements, and medications were grouped as frequently (“once a week,” “2–6 times a week,” and “every day”) and infrequently (“less than once a month,” “1–3 times a month,” and “never”). Although participants were able to select either “exacerbation,” “improvement,” or “no change” for the self-reported outcome of IP in the previous 12 months, only data from exacerbation and improvement were used during analysis. Practitioners were categorized as “medical practitioners” (integrative medicine practitioners, general practitioners, and gastroenterologists) and “healthcare practitioners” (all practitioners).

Analysis and interpretation of the data collected from the PWI-A scale were undertaken according to a previously published work.^24^ Participants who answered consistently 0/10 or 10/10 across all PWI domains were excluded due to a risk of response bias.^24^ For analysis, the raw scores were transformed to a 0–100 scale. The combined mean score from the seven domains represents the participants' overall SWB. A two-sample *t*-test was used to compare the normative mean of the surveyed sample and the Australian population.^25^

The analysis and interpretation of the SF-20 were undertaken according to a previously published work.^22^ For analysis, the SF-20 item scores were transformed to a scale of 0 to 100, with 0 representing the worst perceived health-related outcome. Item scores for each domain were combined and averaged to produce the final domain score (0–100). Higher scores reflect better perceived health-related outcomes, except for bodily pain where a higher score indicates more bodily pain.

## Results

### Demographic characteristics

There were 982 responses to the survey, of which 393 responses were excluded as the initial eligibility questions were not answered and thereby classified as not meeting the eligibility criteria; this left a total of 589 participants. Most participants were female (93%), with a mean age of 45.0 years (SD = 12.1; range 18–82) and a mean BMI of 27.0 (SD = 6.9). Participants' BMI were classified as healthy weight (46.1%), obese (26.9%), overweight (23.8%), and underweight (3.3%). Most participants were born in Australia (81.0%) and resided in New South Wales (29.7%), Queensland (27.3%), Victoria (17.5%), or Western Australia (10.7%), in an urban (70.6%), rural (27.3%), or remote area (2.0%). Most participants described their income manageability as “easy or not too bad” (46.5%), followed by “difficult some of the time” (32.3%) or “difficult all the time” (21.2%). The major health concerns reported by participants were IP (*n* = 300, 50.9%), autoimmune conditions (*n* = 40, 6.8%), Hashimoto's thyroiditis (*n* = 28, 4.8%), gastrointestinal issues (*n* = 24, 4.1%), chronic fatigue syndrome (*n* = 21, 3.6%), and rheumatoid arthritis (*n* = 18, 3.1%).

### Practitioners consulted with, and treatments used, for managing increased IP

Participants most frequently reported using dietary products (87.9%), dietary supplements (72.9%), and lifestyle therapies (54.6%) for the management of IP. Medications were infrequently used by participants for the treatment of IP (8.5%). Self-prescribing of treatment methods for the management of IP was most frequently reported (59.6%), followed by prescription from a naturopath (43.1%), integrative medicine practitioner (19.3%), general practitioner (16.8%), and nutritionist (12.4%) (Table 1). Both dietary products (53.0%) and lifestyle therapies (33.8%) were frequently self-prescribed. However, dietary supplements and medications were most frequently prescribed by a naturopath (37.3%) and general practitioner (4.4%), respectively.

**Table 1.** Frequency of Treatment Methods Used for Increased Intestinal Permeability (*n* = 483)

Who prescribed treatment	Treatment methods used for increased IP^a^									
	Total		Dietary products		Lifestyle therapies		Dietary supplements		Medications	
	*n*	%	*n*	%	*n*	%	*n*	%	*n*	%
Self-prescribed	288	59.6	256	53.0	163	33.8	156	32.3	11	2.3
Naturopath	208	43.1	175	36.2	104	21.5	180	37.3	0	0.0
Integrative medicine practitioner	93	19.3	77	15.9	47	9.7	84	17.4	11	2.3
General practitioner	81	16.8	60	12.4	30	6.2	34	7.0	21	4.4
Nutritionist	60	12.4	57	11.8	28	5.8	47	9.7	0	0.0
Dietitian	37	7.7	34	7.0	12	2.5	15	3.1	0	0.0
Chinese medicine practitioner	28	5.8	17	3.5	12	2.5	24	5.0	0	0.0
Chiropractor	28	5.8	16	3.3	22	4.6	15	3.1	0	0.0
Acupuncturist	24	5.0	12	2.5	11	2.3	18	3.7	0	0.0
Herbalist	24	5.0	15	3.1	14	2.9	22	4.6	0	0.0
Gastroenterologist	20	4.1	13	2.7	5	1.0	8	1.7	3	0.6
Kinesiologist	20	4.1	14	2.9	12	2.5	12	2.5	0	0.0
Ayurvedic practitioner	12	2.5	10	2.1	5	1.0	9	1.9	0	0.0
Homeopath	12	2.5	8	1.7	7	1.5	9	1.9	0	0.0
Osteopath	7	1.5	6	1.2	5	1.0	5	1.0	0	0.0
Pharmacist	5	1.0	1	0.2	1	0.2	5	1.0	1	0.2
Nurse	4	0.8	4	0.8	2	0.4	2	0.4	2	0.4
Nurse practitioner	2	0.4	2	0.4	2	0.4	1	0.2	1	0.2

^a^ Participants were able to select multiple treatment methods.

IP, intestinal permeability.

### Self-reported outcome of increased IP

In the previous 12 months, more participants reported that their IP had improved (55.8%). Half of the participants (50.0%) reported that IP affected their daily living 7 days a week. Further, participants who described an improvement in their IP during the previous 12 months reported that IP affected their daily life 4.0 days a week (95% CI: 3.6–4.4); however, participants who described exacerbation of their IP in the previous 12 months reported that IP affected their daily life 6.0 days a week (95% CI: 5.7–6.3; *p* < 0.001).

A self-reported improvement in IP was associated with participants who were treated by a practitioner compared with those who were not treated by a practitioner (76.1% vs. 23.9%; *p* < 0.001). Participants who reported that their IP had worsened in the previous 12 months had a significantly higher mean BMI compared with those who reported an improvement in their IP in the past 12 months (28.4 vs. 25.5; *p* < 0.001). Multivariate logistic regression analysis found that the use of NSAIDs (*β* = 1.08; 95% CI: 0.17–1.98; *p* = 0.021), lifestyle therapies (*β* = 1.08; 95% CI: 0.46–1.70; *p* = 0.001), and *Saccharomyces boulardii* (*β* = 1.56; 95% CI: 0.46–2.67; *p* = 0.006) were predictors of a greater number of days each week that IP was reported to affect daily living. However, reporting an improvement of their IP in the previous 12 months (*β* = −1.78; 95% CI: −2.39 to −1.17; *p* < 0.001), and infrequently (*β* = −0.90; 95% CI: −1.64 to −0.16; *p* = 0.017) or frequently (*β* = −0.82; 95% CI: −1.49 to −0.16; *p* = 0.016) practicing yoga were found to be predictors for a fewer number of days affecting daily living each week.

### Treatment-related characteristics of increased IP improvement

Participants who reported an improvement in their IP in the previous 12 months were more likely to be treated by a health care practitioner (OR = 2.04, *p* = 0.015), use dietary supplements (OR = 2.66, *p* = 0.003), participate in vigorous exercise (OR = 2.99, *p* < 0.001), and employ vagus nerve stimulation (OR = 3.10, *p* = 0.010) (Table 2). Further, participants who reported an improvement in their IP during the previous 12 months were also less likely to consume gluten (OR = 0.35, *p* < 0.001) or use NSAIDs (OR = 0.35, *p* = 0.022).

**Table 2.** Treatment-Related Characteristics and the Improvement of Increased Intestinal Permeability in the Previous 12 Months (*n* = 287)

Characteristics	Odds ratio (95% CI)	*p*
Treating person		
Self	1.00	
Health care practitioner	2.04 (1.15–3.61)	0.015
Gluten		
Never	1.00	
Frequently	0.35 (0.20–0.61)	<0.001
Vigorous exercise		
Never	1.00	
Frequently	2.99 (1.61–5.53)	<0.001
Vagus nerve stimulation		
Never	1.00	
Frequently	3.10 (1.31–7.31)	0.010
NSAIDs		
Never	1.00	
Infrequently	0.48 (0.26–0.86)	0.014
Frequently	0.35 (0.15–0.86)	0.022
Using dietary supplements		
No	1.00	
Yes	2.66 (1.40–5.05)	0.003

NSAIDs, nonsteroidal anti-inflammatory drugs.

### Associations between common dietary products, lifestyle therapies, medications, and the self-reported outcome of increased IP

Participants who reported frequently consuming organic foods (*p* < 0.001), fermented foods (*p* = 0.004), bone broth (*p* = 0.001), collagen (*p* < 0.001), or apple cider vinegar (*p* = 0.026) described an improvement in their IP in the previous 12 months compared with those who infrequently consumed these dietary products (Table 3). Further, participants who reported infrequently consuming dairy products (*p* = 0.012), refined sugar (*p* < 0.001), or gluten-containing products (*p* < 0.001) described an improvement in their IP in the previous 12 months compared with participants who reported frequently consuming these dietary products. Participants who reported frequently practicing breathing exercises (*p* < 0.001), stress management (*p* < 0.001), meditation (*p* = 0.037), vigorous exercise (*p* < 0.001), yoga (*p* = 0.001), or vagus nerve stimulation (*p* < 0.001) more commonly described an improvement in their IP in the previous 12 months compared with participants who infrequently reported practicing these lifestyle therapies. Participants who infrequently used NSAIDs (*p* = 0.001) more commonly described an improvement in their IP in the previous 12 months compared with participants who frequently used NSAIDs.

**Table 3.** Associations Between Common Dietary Products, Lifestyle Therapies, Medications, and the Self-Reported Outcome of Intestinal Permeability in the Previous 12 Months (*n* = 483)

			Self-reported outcome of increased IP in the previous 12 months		
	Total		Exacerbation	Improvement	
	*n*	%	%	%	*p*
Dietary products					
Red meat					
Frequently	357	74.2	44.6	55.4	0.909
Infrequently	124	25.8	43.9	56.1	
Organic foods					
Frequently	331	69.4	37.6	62.4	<0.001
Infrequently	146	30.6	58.8	41.2	
Dairy					
Frequently	279	57.9	50.6	49.4	0.012
Infrequently	203	42.1	36.4	63.6	
Refined sugar					
Frequently	239	49.8	59.6	40.4	<0.001
Infrequently	241	50.2	30.8	69.2	
Fermented foods					
Frequently	220	46.2	36.1	63.9	0.004
Infrequently	256	53.8	52.3	47.7	
Gluten					
Frequently	213	44.4	64.1	35.9	<0.001
Infrequently	267	55.6	30.3	69.7	
Apple cider vinegar					
Frequently	179	37.3	36.1	63.9	0.026
Infrequently	301	62.7	49.0	51.0	
Bone broth					
Frequently	173	36.1	32.5	67.5	0.001
Infrequently	306	63.9	51.3	48.7	
Collagen					
Frequently	166	35.0	31.1	68.9	<0.001
Infrequently	308	65.0	51.3	48.7	
Alcohol					
Frequently	148	30.9	47.4	52.6	0.472
Infrequently	331	69.1	43.1	56.9	
Lifestyle therapies					
Breathing exercises					
Frequently	212	45.5	34.4	65.6	<0.001
Infrequently	254	54.5	55.3	44.7	
Stress management					
Frequently	210	45.2	34.0	66.0	<0.001
Infrequently	255	54.8	57.1	42.9	
Meditation					
Frequently	191	40.8	38.7	61.3	0.037
Infrequently	277	59.2	50.6	49.4	
Vigorous exercise					
Frequently	146	30.9	28.4	71.6	<0.001
Infrequently	327	69.1	51.4	48.6	
Yoga					
Frequently	133	28.5	31.3	68.8	0.001
Infrequently	333	71.5	50.7	49.3	
Vagus nerve stimulation					
Frequently	61	13.3	20.5	79.6	<0.001
Infrequently	399	86.7	48.8	51.2	
Medications					
NSAIDs					
Frequently	63	13.4	69.2	30.8	0.001
Infrequently	407	86.6	40.2	59.8	
Prednisone					
Frequently	16	3.5	41.7	58.3	0.827
Infrequently	447	96.5	44.9	55.1	
Methotrexate					
Frequently	11	2.4	57.1	42.9	0.704
Infrequently	448	97.6	43.9	56.1	
Antibiotics					
Frequently	6	1.3	60.0	40.0	0.657
Infrequently	460	98.7	43.6	56.4	

### Frequency of dietary supplements use for the treatment of increased IP

The most frequently used dietary supplements for the management of IP were probiotics (36.1%), herbal mixtures (26.6%), prebiotics (21.7%), zinc (21.7%), glutamine (19.4%), magnesium (19.1%), and vitamin D (15.6%) (Table 4). Dietary supplements were most frequently used by participants who described an improvement in their IP during the previous 12 months compared with those who described exacerbation of their IP (63.3%–86.8% vs. 13.2%–36.7%). Participants frequently reported using dietary supplements as prescribed by a practitioner rather than self-prescribed (66.7%–87.8% vs. 12.2%–33.3%) (Table 4). There was a statistically significant association between the use of dietary supplements and the self-reported outcome of IP. Specifically, participants who used zinc (*p* = 0.05), glutamine (*p* = 0.02), magnesium (*p* = 0.006), vitamin C (*p* = 0.03), or vitamin B complex (*p* = 0.001) described an improvement in their IP during the previous 12 months.

**Table 4.** Associations Between Dietary Supplements and Self-reported Outcome of Increased Intestinal Permeability in the Previous 12 Months and Percentage for Person Prescribing Each Treatment (*n* = 346)

Dietary supplements	Self-reported outcome of IP in the previous 12 months					Person who prescribed treatment	
	Total		Exacerbation	Improvement		Self-prescribed	Practitioner prescribed
	*n*	%	%	%	*p*	%	%
Probiotic	125	36.1	33.3	66.7	0.483	27.0	73.0
Herbal mixtures	92	26.6	28.4	71.6	0.111	26.4	73.6
Prebiotic	75	21.7	27.6	72.4	0.113	23.0	77.0
Zinc	75	21.7	25.4	74.6	0.05	20.0	80.0
Glutamine	67	19.4	22.5	77.6	0.02	25.8	74.2
Magnesium	66	19.1	19.2	80.9	0.006	29.2	70.8
Vitamin D	54	15.6	35.9	64.1	0.956	30.2	69.8
Vitamin C	50	14.5	18.8	81.3	0.03	20.4	79.6
Vitamin B complex	49	14.2	13.2	86.8	0.001	12.2	87.8
Omega 3	48	13.9	33.3	66.7	0.689	33.3	66.7
Curcuma longa	42	12.1	23.3	76.7	0.114	30.9	69.1
Slippery elm	41	11.9	28.6	71.4	0.366	17.1	82.9
Aloe vera	39	11.3	24.1	75.9	0.146	23.7	76.3
Digestive enzyme	37	10.7	36.7	63.3	0.963	13.5	86.5
Multivitamin	37	10.7	20.7	79.3	0.062	24.3	75.7
Amino acid mix	31	9.0	32.0	68.0	0.637	29.0	71.0
Saccharomyces boulardii	21	6.1	18.8	81.3	0.131	14.3	85.7
Vitamin A	19	5.5	33.3	66.7	0.806	26.3	73.7

### Subjective well-being and HRQoL

There was a statistically significant difference in overall SWB and each domain of SWB between Australian adults with suspected IP and the Australian population (*p* < 0.001). Specifically, Australian adults with suspected IP had lower (i.e., worse) average scores for all domains compared with the Australian population. A *t*-test showed that participants who described exacerbation of their IP had a worse (*M* = 54.7, SD = 20.3) SWB than those reporting an improvement (*M* = 66.1, SD = 19.6) in their IP (*p* < 0.001). Spearman's correlation analysis revealed that the number of days that IP affects daily life had a negative correlation with SWB and HRQoL (*p* < 0.001). Results for correlation analyses are summarized in Table 5.

**Table 5.** Spearman's Correlation Between Quality of Life and Subjective Well-being with the Number of Days Increased Intestinal Permeability Affects Daily Life Each Week (0–7 Days)

	*n*	Mean	SD	Correlation coefficient	*p*
Subjective well-being					
Personal well-being index	422	60.3	20.3	−0.402	<0.001
Standard of living	422	65.0	25.5	−0.313	<0.001
Health	422	43.4	24.6	−0.453	<0.001
Achieving in life	422	56.1	25.6	−0.377	<0.001
Personal relationship	422	64.2	26.3	−0.261	<0.001
Personal safety	422	75.3	24.3	−0.193	<0.001
Community connectedness	422	59.3	27.2	−0.277	<0.001
Future security	422	58.8	27.9	−0.273	<0.001
Quality of life					
Physical functioning	423	61.9	33.8	−0.275	<0.001
Role functioning	423	57.3	42.5	−0.335	<0.001
Social functioning	423	60.5	32.3	−0.388	<0.001
Mental health	423	55.0	21.6	−0.294	<0.001
Health perception	423	37.2	28.5	−0.474	<0.001
Bodily pain	423	50.4	25.1	0.316	<0.001

Score ranges from 0 to 100. A high score indicates better health except for pain, where a high score indicates more pain.

SD, standard deviation.

### Subjective well-being and common dietary products, lifestyle therapies, and medications

Pairwise comparison found a statistically significant difference between the overall SWB of participants, and the frequency of common dietary products, lifestyle therapies, and medication use. Participants who used any form of dietary products (*M* = 61.0, SD = 20.4) for the treatment of IP were found to have better SWB compared with those who never used dietary products (*M* = 54.6, SD = 18.7) (*p* = 0.023). Further, participants who never consumed gluten-containing foods (*M* = 65.2, SD = 21.5) were found to have better SWB compared with participants who frequently consumed gluten (*M* = 59.1, SD = 19.8) (*p* = 0.037). However, participants who frequently consumed alcohol (*M* = 64.9, SD = 18.7) were found to have better SWB compared with those who never consumed alcohol (*M* = 54.0, SD = 22.4) (*p* < 0.001). Further, participants who frequently practiced breathing exercises (*M* = 63.0, SD = 19.4; *M* = 56.2, SD = 21.5) (*p* = 0.014), stress management (*M* = 62.4, SD = 19.0; *M* = 56.1, SD = 23.7) (*p* = 0.036), vigorous exercise (*M* = 66.2, SD = 18.4; *M* = 55.3, SD = 20.9) (*p* < 0.001), or yoga (*M* = 68.0, SD = 17.0; *M* = 56.0, SD = 20.9) (*p* < 0.001) were found to have better SWB compared with participants who never participated in these lifestyle therapies. Lastly, participants who never used NSAIDs (*M* = 62.5, SD = 21.5) were found to have better SWB compared with those who frequently used them (*M* = 54.3, SD = 19.5) (*p* = 0.026).

### Multiple regression predicting SWB and HRQoL

Seven regression models predicting overall SWB, and each HRQoL domain were undertaken. The results of these regression models found that the outcome of IP in the previous 12 months, BMI, the treating person, and the use of dietary products and lifestyle therapies were all statistically significant predictors of overall SWB and each HRQoL domain (Table 6). Specifically, improvement of IP (*β* = 10.70, *p* < 0.001) and using dietary products (*β* = 12.12, *p* = 0.008) were predictors of better SWB whereas being obese (*β* = −5.70, *p* = 0.035), treated by a medical practitioner (*β* = −6.35, *p* = 0.016), and using lifestyle therapies (*β* = −6.30, *p* = 0.010) were predictors of worse SWB. Regarding HRQoL, all domains except physical functioning saw improvement in IP as a statistically significant predictor for higher HRQoL (Table 6).

**Table 6.** Multiple Regression Predicting Subjective Well-being and Health-Related Quality of Life

		Health-related quality of life					
	Subjective wellbeing (*n* = 301),* β *(95% CI),* p *value	Physical functioning (*n* = 417),* β *(95% CI),* p *value	Role functioning (*n* = 306),* β *(95% CI),* p *value	Social functioning (*n* = 306),* β *(95% CI),* p *value	Mental health (*n* = 304),* β *(95% CI),* p *value	Health perception (*n* = 302),* β *(95% CI),* p *value	Bodily pain (*n* = 304),* β *(95% CI),* p *value
Improvement of IP in previous 12 months	10.70 (6.01 to 15.39), <0.001		21.06 (11.60 to 30.51), <0.001	18.83 (11.72 to 25.94), <0.001	10.57 (5.56 to 15.58), <0.001	21.88 (15.76 to 27.99), <0.001	−11.74 (−17.53 to −5.94), <0.001
Using dietary products	12.12 (3.21 to 21.03), 0.008				15.79 (6.24 to 25.33), 0.001		
Using lifestyle therapies	−6.30 (−11.05 to −1.54), 0.010		−14.97 (−24.59 to −5.35), 0.002	−9.30 (−16.53 to −2.07), 0.012	−7.30 (−12.36 to −2.23), 0.005	−7.45 (−13.58 to −1.33), 0.017	5.86 (0.07 to 11.64), 0.047
BMI							
Normal weight	1.00	1.00			1.00	1.00	1.00
Obese	−5.70 (−10.99 to −0.41), 0.035	−15.51 (−22.59 to −8.43), <0.001			−5.91 (−11.58 to −0.24), 0.041	−12.89 (−19.88 to −5.91), <0.001	12.69 (6.05 to 19.33), <0.001
Treating person							
Self	1.00	1.00				1.00	
Medical practitioner	−6.35 (−11.52 to −1.18), 0.016	−13.06 (−20.46 to −5.66), 0.001				−9.76 (−16.57 to −2.95), 0.005	

BMI, body mass index; CI, confidence interval.

## Discussion

**FIG. 1.**
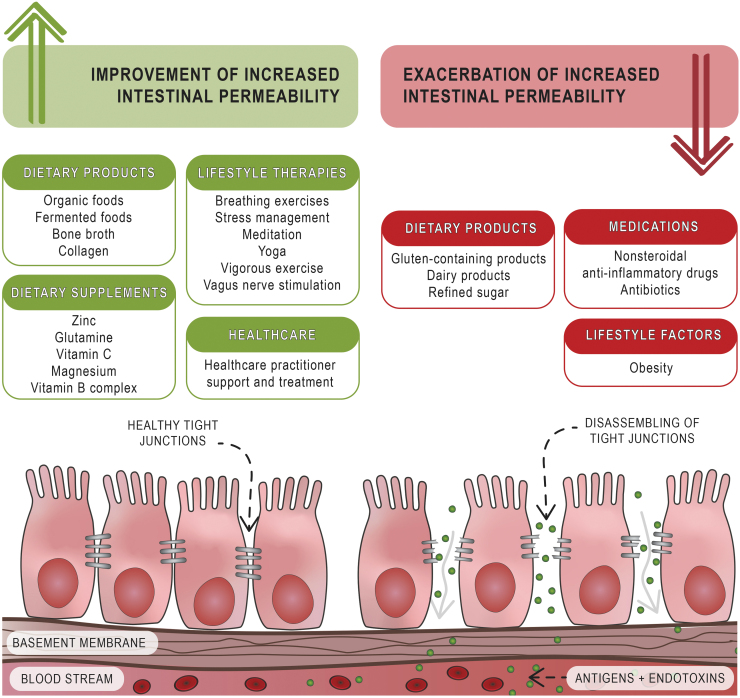
Participant's characteristics found to be associated with the improvement and exacerbation of increased intestinal permeability.

This study is the first to explore the HRQoL and SWB of Australian adults with suspected IP. Our results suggest that altered IP may pose a greater health burden than previously thought, providing the first indication that Australian adults with altered IP are susceptible to poor SWB and HRQoL. Further, several participant characteristics were found to be associated with the improvement or exacerbation of IP (Fig. 1).

### Increased IP and SWB and HRQoL

Our results suggest that Australian adults with suspected IP have a lower SWB compared with the Australian population. Further, improvement in IP is suggested to be a significant predictor of SWB and HRQoL. These results provide the first indication that a relationship between both SWB and HRQoL and altered IP exists in a diverse range of health conditions. In support of this relationship, Australian adults with gastrointestinal disorders (many of which are associated with altered IP)^4^ have been found to have a lower HRQoL compared with Australian adults without gastrointestinal disorders.^26^ Further, a lower QoL has been reported in diarrhea-predominant irritable bowel syndrome patients with IP compared with those with a normal intestinal integrity.^27^ The association between altered IP and both SWB and HRQoL contributes to a much needed clinical understanding of altered IP, especially as the consequence and clinical relevance of altered IP in clinical practice have recently been questioned.^12^ Further, the correlation found between both SWB and HRQoL and the number of days that IP affects daily living suggests that the previously reported symptoms^7^ and biomarkers^5^ of altered IP are not the only clinical consequence of altered IP, with both SWB and HRQoL now suggested to be involved.

### Health care and increased IP

The care provided by health care practitioners compared with self-care differs not only in the treatments used but also in the reported outcomes. First, this study identified a high prevalence of self-prescription of treatment interventions, primarily dietary products, and lifestyle therapies, for the management of IP. Dietary supplements and medication were most frequently prescribed by a health care practitioner. These findings coincide with existing research that suggests that complementary and integrative medicine practitioners frequently use dietary supplements while also using a multimodal and personalized approach for the management of altered IP.^9^ Working alongside a health care practitioner has also been suggested to provide greater health outcomes compared with no clinic-based support.^28–30^ This may explain why in this study Australian adults who report an improvement in their IP are two times more likely to be treated by a health care practitioner. Second, our study found that only 24% of self-treated participants reported an improvement in their IP compared with 76% of practitioner-treated participants. These findings suggest that the care provided by health care practitioners to Australian adults with suspected IP may have beneficial effects on the outcomes of altered IP. Further, health care practitioners, especially those with limited experience in the management of altered IP, may draw on the findings of this study to gain a deeper understanding as to the treatment methods used by Australian adults with IP.

### Features associated with increased IP improvement

Participants who reported an improvement in their IP were 35% less likely to consume gluten or use NSAIDs. Our results also found that participants who indicated that they avoided consuming gluten-containing foods and never used NSAIDs were associated with a better SWB. These results concur with clinical studies that show that the consumption of gluten-containing products and the use of NSAIDs induce IP.^31,32^ Practitioners who treat IP also advocate for their patients with IP to avoid gluten and NSAIDs.^9^

The finding that vitamin C and vigorous exercise is associated with improvement of IP conflicts with existing research. First, preliminary research suggests that 500 mg of vitamin C (ascorbic acid) may induce a rearrangement of the actin cytoskeleton and thereby an exacerbation of IP.^31,33^ Potentially, the association between vitamin C intake and improvement of IP may be the result of the frequent use in dietary supplements, especially as participants who reported an improvement in their IP were 2.7 times more likely to use dietary supplements. Research has demonstrated a causative link between vigorous exercise and altered IP.^34^ As a result of redistribution of blood flow and splanchnic hypoperfusion during vigorous exercise, damage to mucosal and epithelial cells may occur, thereby paving the way for exacerbation of IP.^34^ The improvement associated with vigorous exercise in our study may be the result of improved health; for example, as health and well-being improve so does the ability to participate in exercise.^35^ Further large-scale trials and epidemiological research is needed to confirm both of these hypotheses.

### Research agenda

Our study provides useful information where further research can draw on the findings to inform clinical trials and clinical practice guidelines. The identified characteristics found to be associated with the improvement and exacerbation of IP warrant further investigation (Fig. 1). Many of these associated features are yet to be investigated for their effect on IP, with clinical research focusing primarily on dietary supplements and dietary products for the treatment of IP. However, there has been limited investigation exploring the effectiveness of lifestyle therapies in the management of IP.^9^ Nevertheless, many of these lifestyle therapies are reported to have beneficial health outcomes in health conditions with a known association with altered IP.^36,37^ These results provide a foundation for future clinical trials where a study exclusively conducted in primary care ensuring a homogenous study population and standardized diagnostic criteria may confirm the results of this study.

The findings from this study may also help to inform the development of a clinical practice guideline for the management of altered IP. By understanding the treatment methods used, the development of recommendations can incorporate the views and preferences of Australian adults with suspected IP to enable relevant and appropriate recommendations for this patient group.

### Limitations

Although this study involved participants with self-reported suspected IP, whether there was a confirmed diagnosis of IP is unknown. However, previous research has shown that people with self-reported irritable bowel syndrome have similar health care utilization and QoL as those with diagnosed IBS.^38^ Many of the health conditions that participants report experiencing are known to be more prevalent in females and are suggested to be associated with IP, which may explain why 93% of participants were female.^4^ Therefore, these results are considered relevant to females who suspect they have IP rather than Australian adults with a confirmed diagnosis of altered IP. The self-reported outcome of IP has the potential for recall bias and may not reflect improvement or exacerbation of IP. Therefore, to confirm the relationship between both SWB and HRQoL and altered IP, a clinical study that measures IP and evaluates both SWB and HRQoL is required. However, this study provides important and novel information, advancing the research agenda on the clinical consequence of altered IP, and suggests potential treatment strategies that are worth investigating.

## Conclusion

The integrity of the small intestine may pose a greater health burden than previously thought, with susceptibility to poor SWB and HRQoL reported in Australian adults with altered IP. Our results strengthen the clinical relevance and consequence of altered IP, providing the first indication that a relationship between both SWB and HRQoL and altered IP exists. Clinical trials may use these findings to further explore the potential use of the treatment interventions used by Australian adults with suspected IP.
